# Supplementary material for: PARG is essential for Polθ-mediated DNA end-joining by removing repressive poly-ADP-ribose marks
Source: Nat Commun. 2024 Jul 11;15:5822. doi: 10.1038/s41467-024-50158-7 (PMC11236980; doi:10.1038/s41467-024-50158-7)
Supplement: Supplementary file 1 — Supplementary Information [file 41467_2024_50158_MOESM1_ESM.pdf]

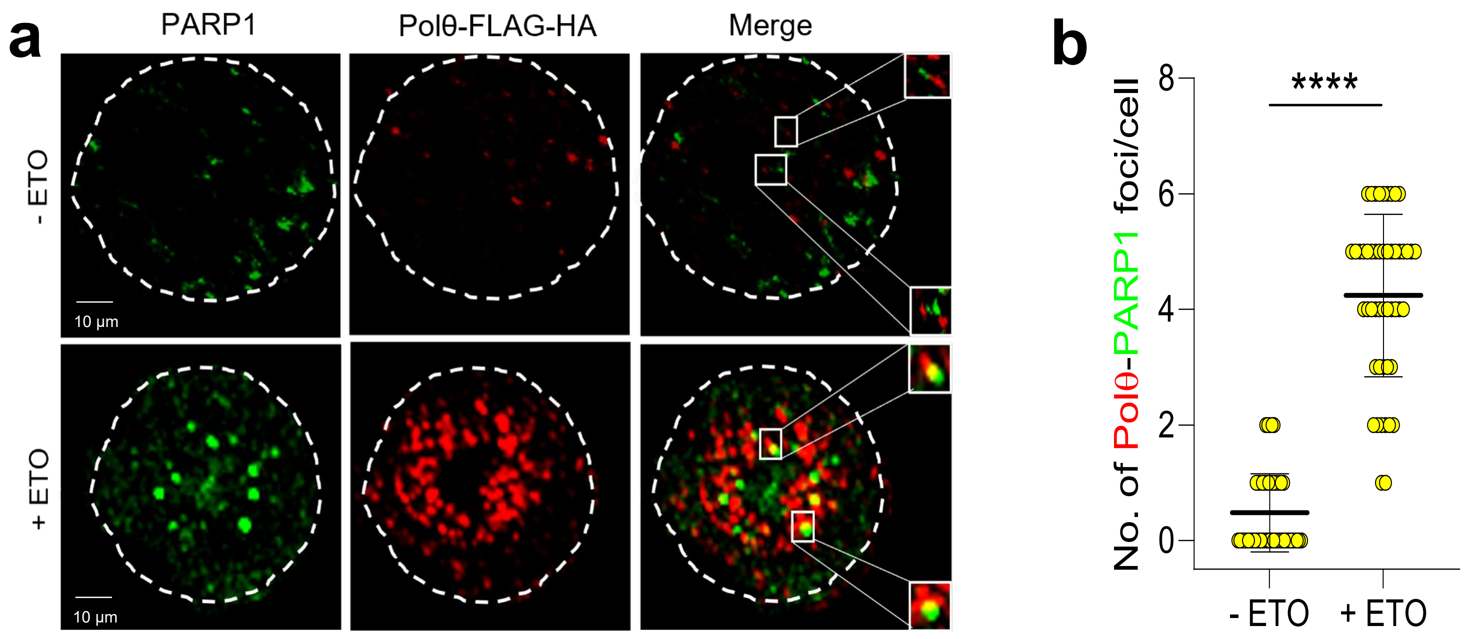

Supplementary Fig. 1. a Confocal microscopic image shows co-localization of Polθ-FLAG-HA and PARP1 in 293T cells overexpressing Polθ-FLAG-HA and exposed to 10Gy Irradiation +/- 25 μM etoposide (ETO). Dimentions:10 μm or 100 nm (for magnified foci). **b** Quantification of Polθ-FLAG-HA foci formation (N=50 cells) from two independent biological replicates. ****p<0.00001 using two-tailed unpaired t-test. Source data are provided as a Source Data file.

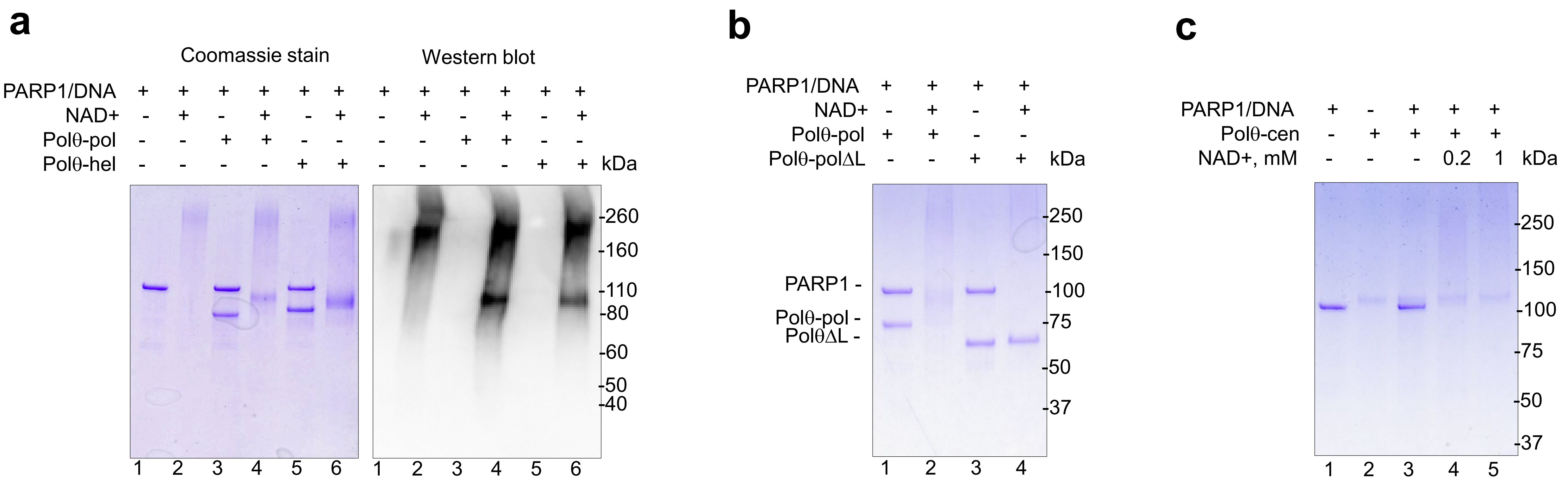

Supplementary Fig. 2. a PARP1 wild-type PARylates Polθ-pol and Polθ-hel *in vitro***.** SDS gel (left) and Western blot (right) showing PARP1 PARylation of Polθ-pol and Polθ-hel *in vitro*. **b** SDS gel showing that PARP1 does not PARylate PolθΔL which lacks 5 disordered domains. **c** SDS gel showing that PARP1 does not PARylate the central domain of Polθ (Polθ-cen).

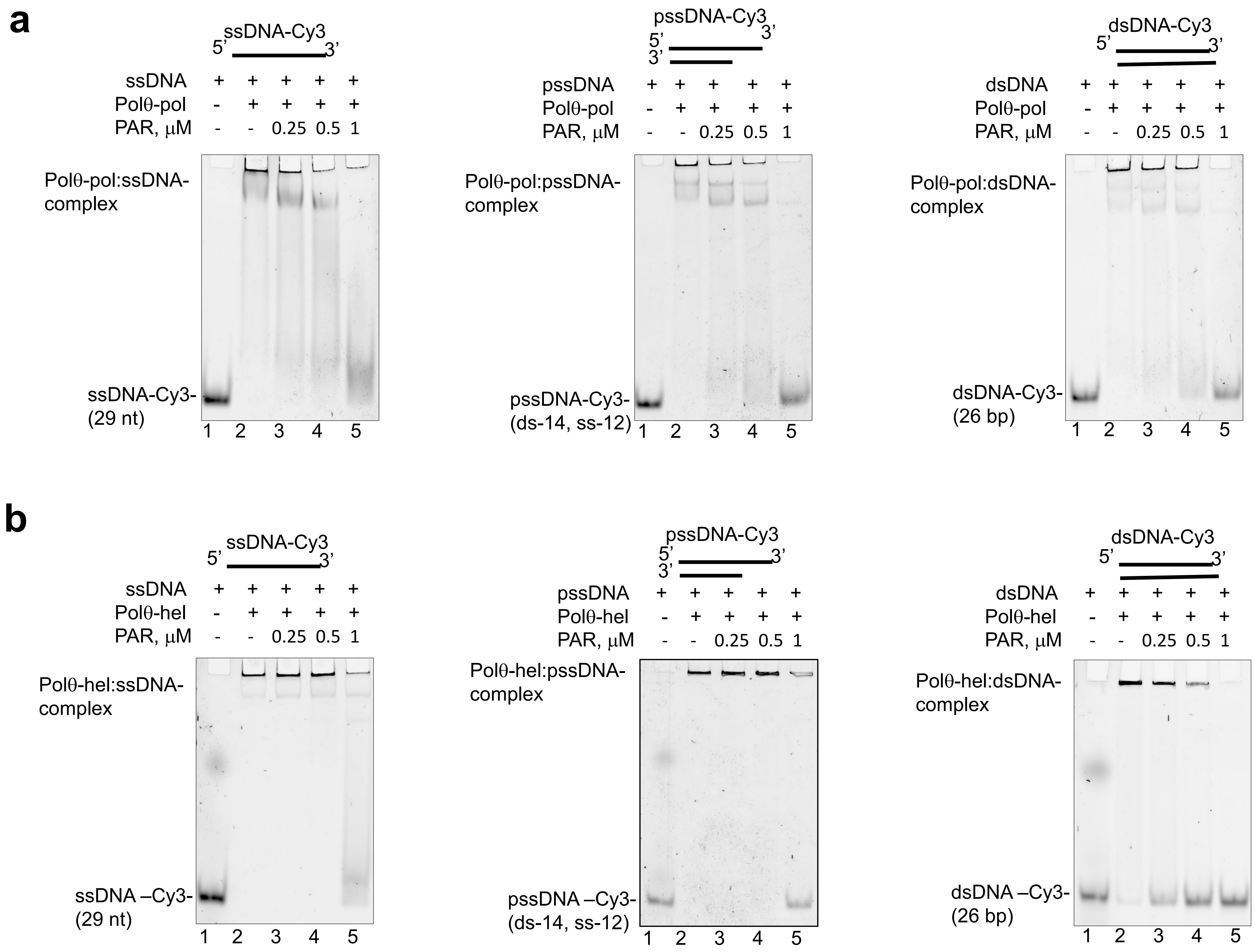

Supplementary Fig. 3. EMSA showing that Polθ-pol (**a**) and Polθ-hel (**b**) are inhibited in DNA binding in the presence of PAR *in trans.* Non-denaturing gels showing Polθ-pol (**a**) and Polθ-hel (**b**) binding of the indicated DNA substrates in the presence and absence of the indicated concentrations of recombinant PAR.

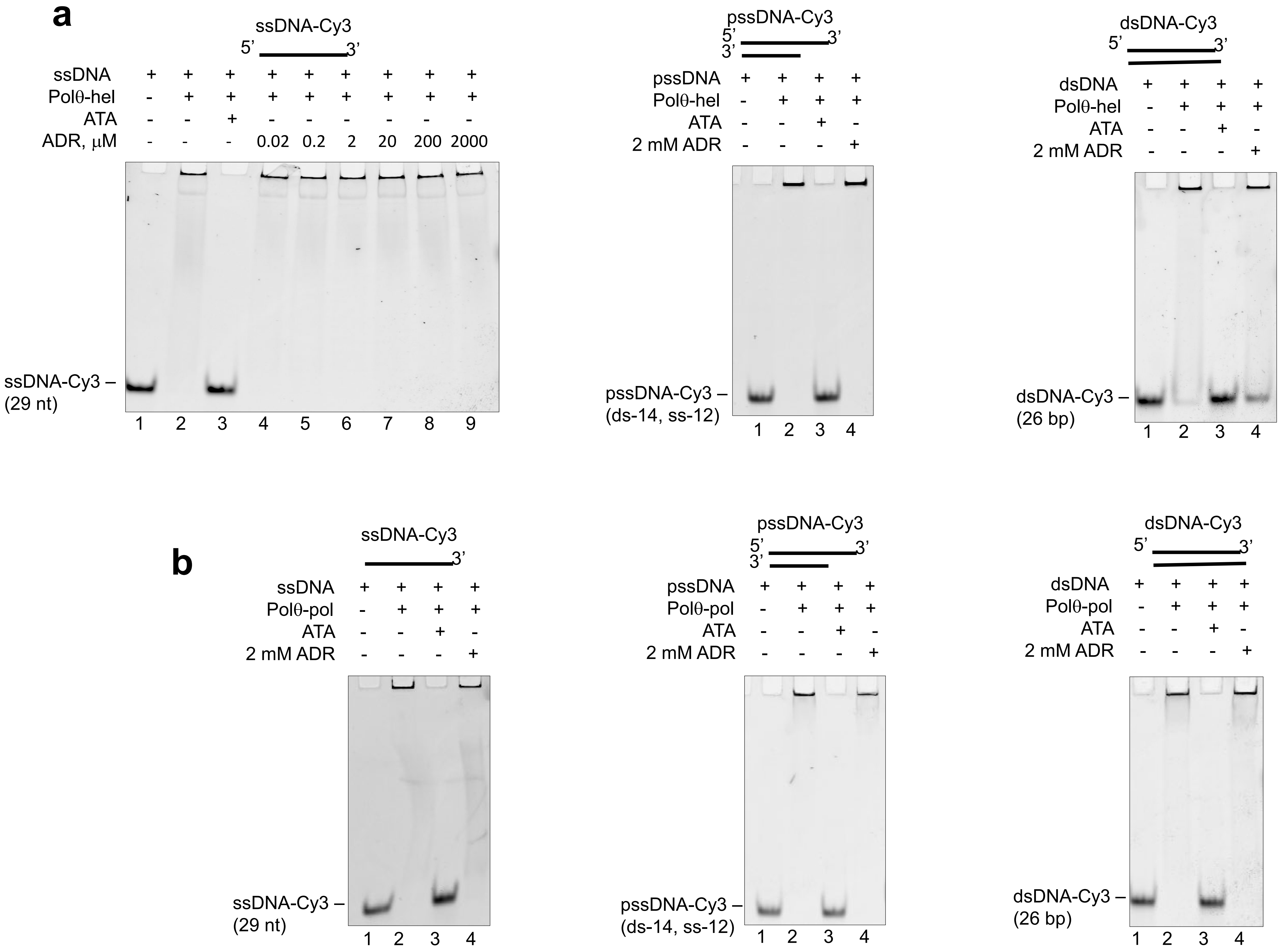

Supplementary Fig. 4. a EMSA showing that Polθ-hel is not significantly inhibited in DNA binding in the presence of ADP-diphosphate ribose (ADR) as a competitor: ssDNA binding (left), pssDNA binding (middle), and dsDNA binding (right). **b** EMSA showing that Polθ-pol is not inhibited in DNA binding in the presence of ADR as a competitor: ssDNA binding (left), pssDNA binding (middle), dsDNA binding (right).

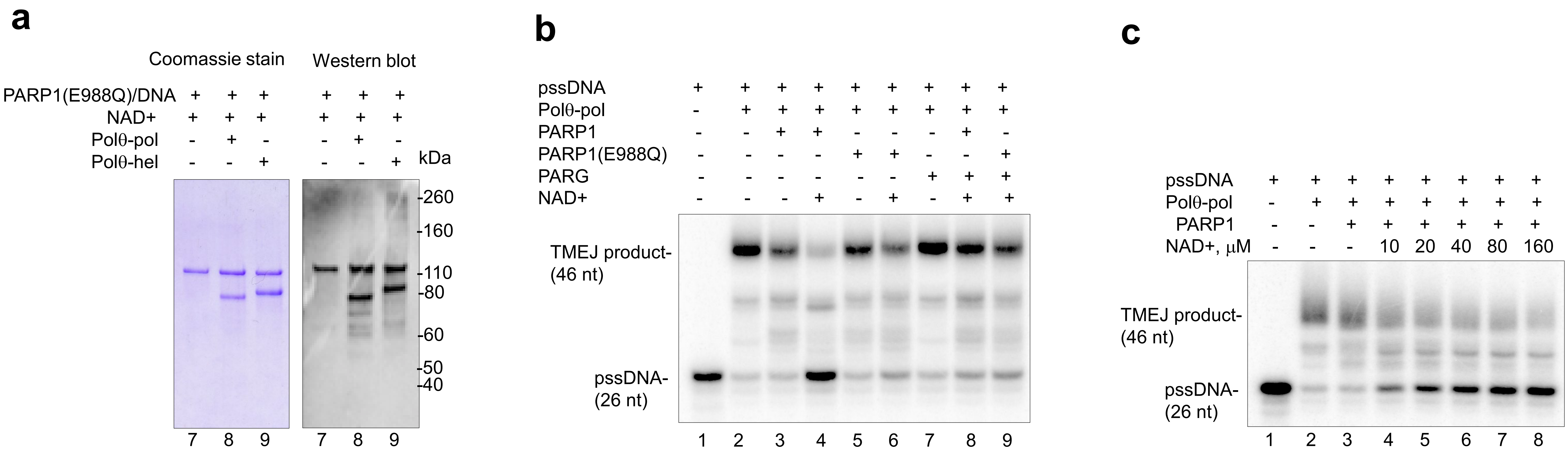

Supplementary Fig. 5. a SDS gel (left) and Western blot (right) showing that PARP1(E988Q) MARylates Polθ-pol and Polθ-hel *in vitro.* **b** Denaturing gel showing that the mutant PARP1(E988Q) only partially inhibits TMEJ activity by Polθ-pol in the presence of NAD+. **c** TMEJ efficiency inhibition depends on the NAD+ concentration.

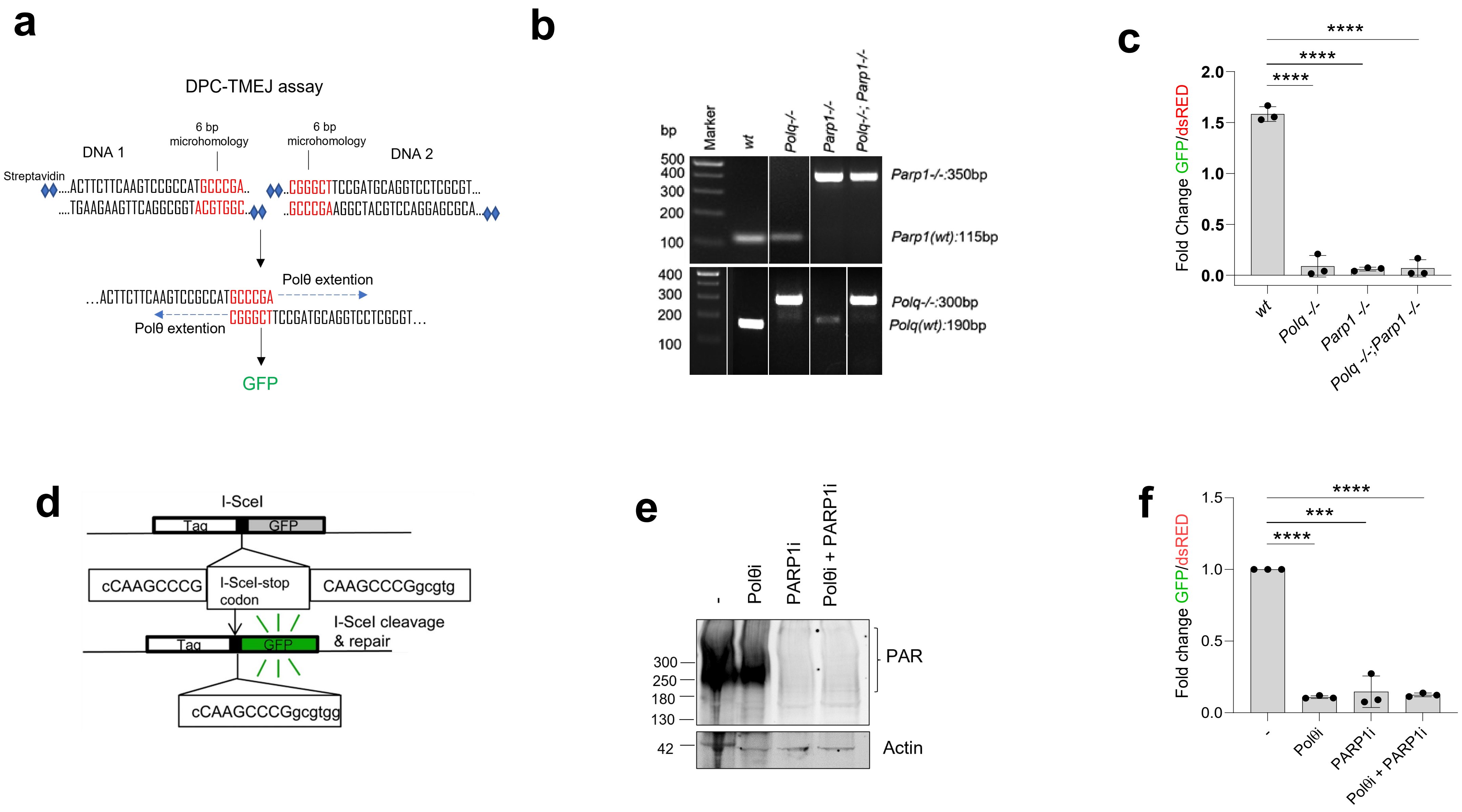

Supplementary Fig. 6. a DPC-TMEJ substrate and the repair of *GFP* cDNA (1). **b** Representative PCR bands of genotyping of mBMCs. **c** DPC-TMEJ activity in the indicated mBMCs co-transfected with DPC-TMEJ reporter (1) and dsRED plasmid. **d** EJ2-GFP cassette integrated into the genome of U2OS cells and the repair of *GFP* cDNA. **e** PARylation detected by Western blot in U2OS cells treated with the indicated inhibitors. **f** TMEJ activity in the U2OS cells co-transfected with I-SceI plasmid and dsRED plasmid and treated with the indicated inhibitors. Results in **c** and **f** represent ratio of GFP+/dsRED+ cells from three independent biological replicates; ****p<0.0001 and ***p<0.001 using one-way ANOVA. Source data are provided as a Source Data file.
.

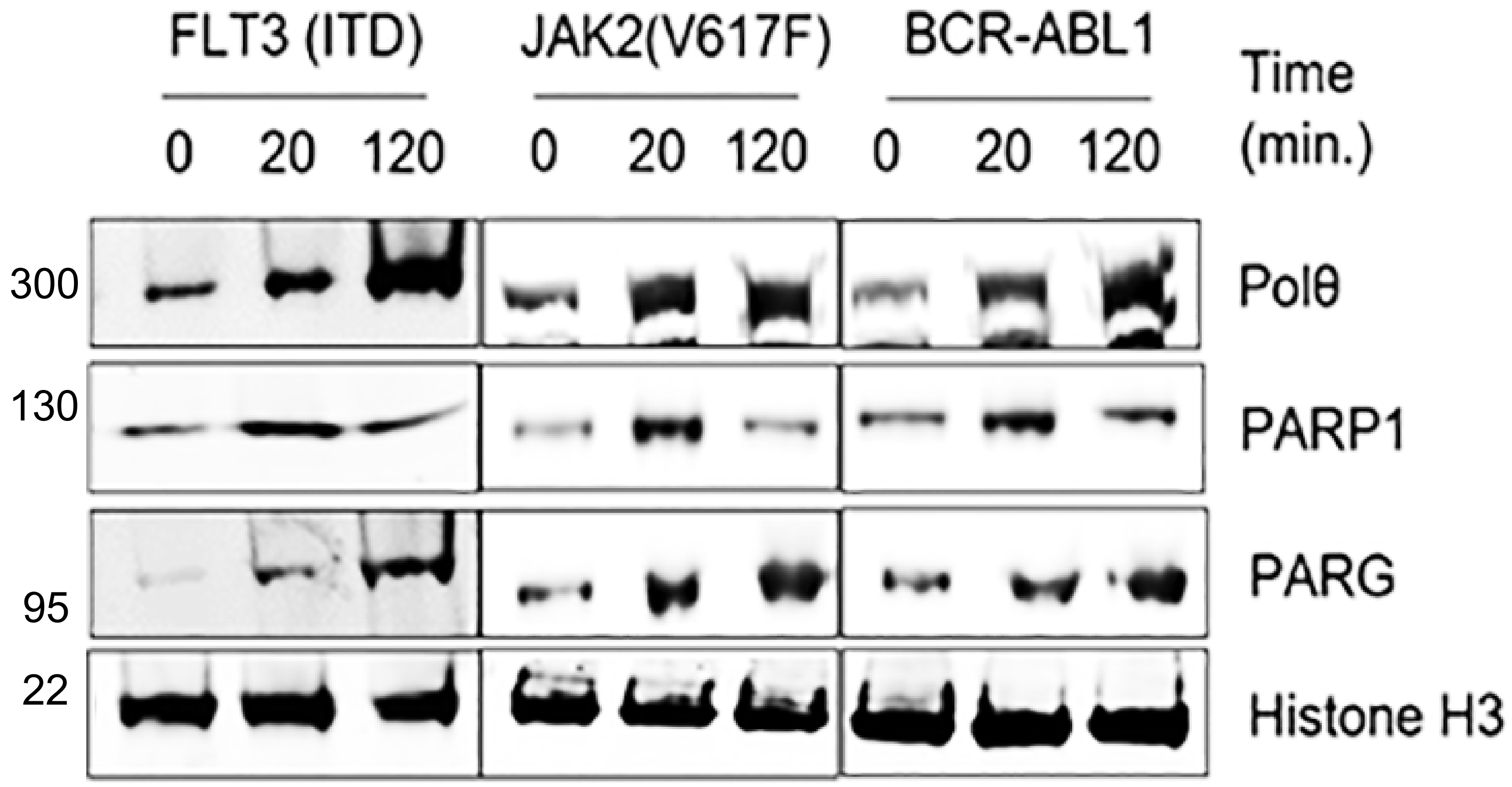

Supplementary Fig. 7. Western blot analysis of the chromatin extracts obtained before (0) and after (20 min, 120 min) irradiation (2Gy) from 32Dcl3 cells expressing the indicated oncogenic tyrosine kinases.

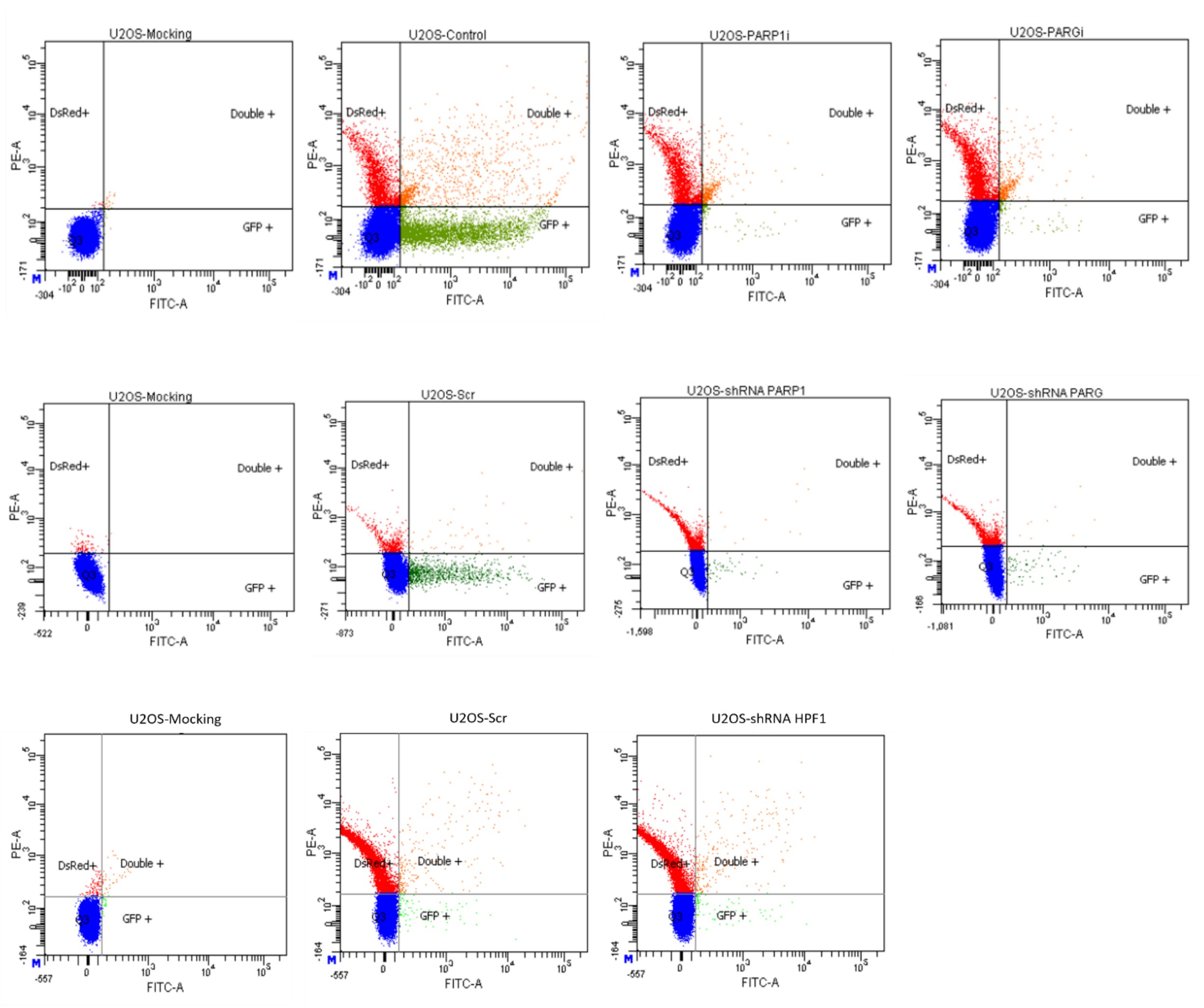

Supplementary Fig. 8. Representative gating detecting GFP+ and dsRED+ cells to assess TMEJ. Experimental details described in Fig. 3 f, h and j.

Supplementary References:

(1) Chandramouly G et al. Polθ promotes the repair of 5'-DNA-protein crosslinks by microhomology-mediated end-joining. Cell reports 2021;34:108820
