# Supplementary material for: PARG is essential for Polθ-mediated DNA end-joining by removing repressive poly-ADP-ribose marks
Source: Nat Commun. 2024 Jul 11;15:5822. doi: 10.1038/s41467-024-50158-7 (PMC11236980; doi:10.1038/s41467-024-50158-7)
Supplement: Supplementary file 3 — Description of Additional Supplementary Files [file 41467_2024_50158_MOESM3_ESM.pdf]

Description of additional Supplementary files

File Name: Supplementary Data 1
Description: KEY RESOURCE TABLE
