# Supplementary material for: PARG is essential for Polθ-mediated DNA end-joining by removing repressive poly-ADP-ribose marks
Source: Nat Commun. 2024 Jul 11;15:5822. doi: 10.1038/s41467-024-50158-7 (PMC11236980; doi:10.1038/s41467-024-50158-7)
Supplement: Supplementary file 5 — Reporting Summary [file 41467_2024_50158_MOESM5_ESM.pdf]

nature portfolio

Corresponding author(s): Tomasz Skorski and Richard Pomerantz

Last updated by author(s): May 29, 2024

Reporting Summary

Nature Portfolio wishes to improve the reproducibility of the work that we publish. This form provides structure for consistency and transparency in reporting. For further information on Nature Portfolio policies, see our Editorial Policies and the Editorial Policy Checklist.

Statistics

For all statistical analyses, confirm that the following items are present in the figure legend, table legend, main text, or Methods section.

n/a	Confirmed	
☐	☒	The exact sample size (*n*) for each experimental group/condition, given as a discrete number and unit of measurement
☐	☒	A statement on whether measurements were taken from distinct samples or whether the same sample was measured repeatedly
☐	☒	The statistical test(s) used AND whether they are one- or two-sided<br>*Only common tests should be described solely by name; describe more complex techniques in the Methods section.*
☒	☐	A description of all covariates tested
☒	☐	A description of any assumptions or corrections, such as tests of normality and adjustment for multiple comparisons
☐	☒	A full description of the statistical parameters including central tendency (e.g. means) or other basic estimates (e.g. regression coefficient) AND variation (e.g. standard deviation) or associated estimates of uncertainty (e.g. confidence intervals)
☐	☒	For null hypothesis testing, the test statistic (e.g. *F*, *t*, *r*) with confidence intervals, effect sizes, degrees of freedom and *P* value noted<br>*Give P values as exact values whenever suitable.*
☒	☐	For Bayesian analysis, information on the choice of priors and Markov chain Monte Carlo settings
☒	☐	For hierarchical and complex designs, identification of the appropriate level for tests and full reporting of outcomes
☒	☐	Estimates of effect sizes (e.g. Cohen's *d*, Pearson's *r*), indicating how they were calculated

Our web collection on statistics for biologists contains articles on many of the points above.

Software and code

Policy information about availability of computer code

Data collection	N/A
Data analysis	Image J 1.53K, GraphPad Prism 8.0.2.263, Odyssey 3.0.30

For manuscripts utilizing custom algorithms or software that are central to the research but not yet described in published literature, software must be made available to editors and reviewers. We strongly encourage code deposition in a community repository (e.g. GitHub). See the Nature Portfolio guidelines for submitting code & software for further information.

Data

Policy information about availability of data

All manuscripts must include a data availability statement. This statement should provide the following information, where applicable:

- Accession codes, unique identifiers, or web links for publicly available datasets
- A description of any restrictions on data availability
- For clinical datasets or third party data, please ensure that the statement adheres to our policy

The sequencing data generated in this study deposited in the figshare. [https://figshare.com/s/8f1254126c624fc79dee]. The mass spectrometry proteomics data have been deposited into the MassIVE (https://massive.ucsd.edu/) and ProteomeXchange (https://proteomecentral.proteomexchange.org/) data repository with the accession number MSV000092839 and PXD045320, respectively. All data used in the analysis are provided within the manuscript and all materials used are described in Supplementary Table 1. Source data are provided with this paper.

Research involving human participants, their data, or biological material

Policy information about studies with human participants or human data. See also policy information about sex, gender (identity/presentation), and sexual orientation and race, ethnicity and racism.

Reporting on sex and gender	N/A
Reporting on race, ethnicity, or other socially relevant groupings	N/A
Population characteristics	N/A
Recruitment	N/A
Ethics oversight	N/A

Note that full information on the approval of the study protocol must also be provided in the manuscript.

Field-specific reporting

Please select the one below that is the best fit for your research. If you are not sure, read the appropriate sections before making your selection.

☒ Life sciences ☐ Behavioural & social sciences ☐ Ecological, evolutionary & environmental sciences

For a reference copy of the document with all sections, see nature.com/documents/nr-reporting-summary-flat.pdf

Life sciences study design

All studies must disclose on these points even when the disclosure is negative.

Sample size	As specified in Materials and Methods and Figure legends.
Data exclusions	No data excluded.
Replication	Data represent biological replicates as indicated in Materials and Methods and Figure legends.
Randomization	N/A
Blinding	All results involving cell lines were obtained from experiments blinded at least one time. Blinding biochemical assays was not possible due to limited personnel and resources.

Reporting for specific materials, systems and methods

We require information from authors about some types of materials, experimental systems and methods used in many studies. Here, indicate whether each material, system or method listed is relevant to your study. If you are not sure if a list item applies to your research, read the appropriate section before selecting a response.

Materials & experimental systems

n/a	Involved in the study	
☐	☒	Antibodies
☐	☒	Eukaryotic cell lines
☒	☐	Palaeontology and archaeology
☐	☒	Animals and other organisms
☒	☐	Clinical data
☒	☐	Dual use research of concern
☒	☐	Plants

Methods

n/a	Involved in the study	
☒	☐	ChIP-seq
☐	☒	Flow cytometry
☒	☐	MRI-based neuroimaging

Antibodies

Antibodies used	As indicated in Materials and Methods.
Validation	By the manufacturers.

Eukaryotic cell lines

Policy information about cell lines and Sex and Gender in Research

Cell line source(s)	As indicated in Materials and Methods.
Authentication	As indicated in Materials and Methods.
Mycoplasma contamination	All used cell line cells were Mycoplasma free.
Commonly misidentified lines (See ICLAC register)	N/A

Animals and other research organisms

Policy information about studies involving animals; ARRIVE guidelines recommended for reporting animal research, and Sex and Gender in Research

Laboratory animals	As indicated in the Materials and Methods.
Wild animals	N/A
Reporting on sex	Sex was not considered in study design.
Field-collected samples	As indicated in the Materials and Methods.
Ethics oversight	As indicated in the Materials and Methods.

Note that full information on the approval of the study protocol must also be provided in the manuscript.

Plants

Seed stocks	N/A
Novel plant genotypes	N/A
Authentication	N/A

Flow Cytometry

Plots

Confirm that:

- ☒ The axis labels state the marker and fluorochrome used (e.g. CD4-FITC).
- ☒ The axis scales are clearly visible. Include numbers along axes only for bottom left plot of group (a 'group' is an analysis of identical markers).
- ☒ All plots are contour plots with outliers or pseudocolor plots.
- ☒ A numerical value for number of cells or percentage (with statistics) is provided.

Methodology

Sample preparation	U2OS cells carrying one copy of E2J-GFP cassette were co-transfected with I-SceI cDNA and dsRED-Mito cDNA (control for transfection efficiency) using lipofectamine 2000 (Invitrogen, Cat No. 11668-500). GFP+ and Red+ cells were analyzed by flow cytometer (Facscanto, BD) followed by Flowjo V.10.10.0 software after transfection 72 h after transfection.
Instrument	Facscanto, BD
Software	Flowjo_V10.10.0
Cell population abundance	N/A

Gating strategy

As indicated in Materials and Methods.

☒ Tick this box to confirm that a figure exemplifying the gating strategy is provided in the Supplementary Information.
