# Supplementary material for: PARG is essential for Polθ-mediated DNA end-joining by removing repressive poly-ADP-ribose marks
Source: Nat Commun. 2024 Jul 11;15:5822. doi: 10.1038/s41467-024-50158-7 (PMC11236980; doi:10.1038/s41467-024-50158-7)
Supplement: Supplementary file 6 — Source Data [file 41467_2024_50158_MOESM6_ESM.zip › Source_data/451052_2_related_ms_9129728_sgcv7d.pptx]

## Slide 1
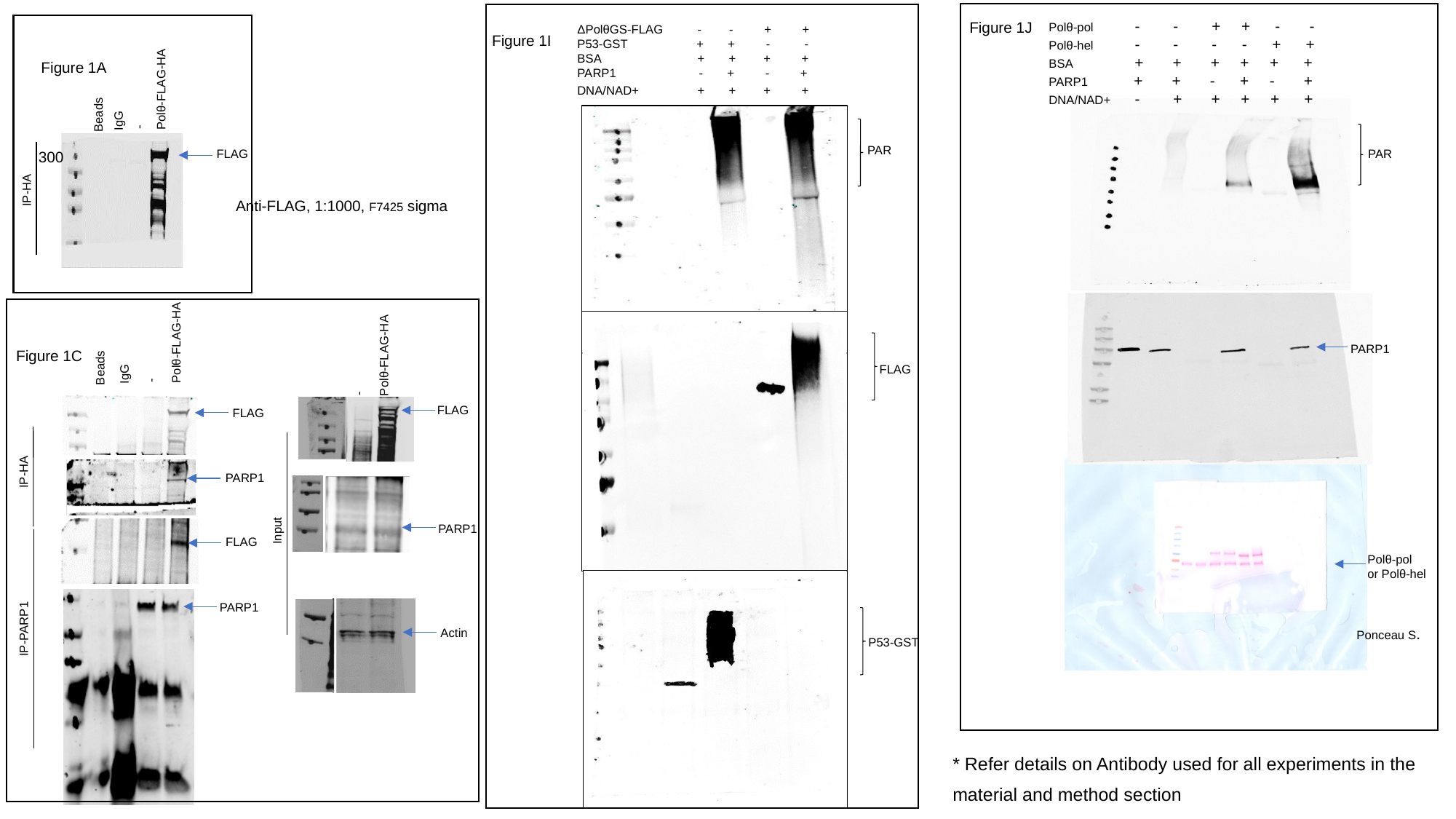

Polθ-pol - - + + - -
Polθ-hel - - - - + +
BSA + + + + + +
PARP1 + + - + - +
DNA/NAD+ - + + + + +
PAR
PARP1
Ponceau S.
Figure 1J
ΔPolθGS-FLAG - - + +
P53-GST + + - -
BSA + + + +
PARP1 - + - +
DNA/NAD+ + + + +
PAR
FLAG
P53-GST
Figure 1I
Polθ-FLAG-HA
-
IgG
Beads
FLAG
300
IP-HA
Figure 1A
Anti-FLAG, 1:1000, F7425 sigma
Polθ-FLAG-HA
Figure 1C
Polθ-FLAG-HA
-
IgG
Beads
-
FLAG
FLAG
PARP1
PARP1
IP-PARP1
Actin
IP-HA
Input
FLAG
Polθ-pol
or Polθ-hel
PARP1
* Refer details on Antibody used for all experiments in the material and method section

## Slide 2
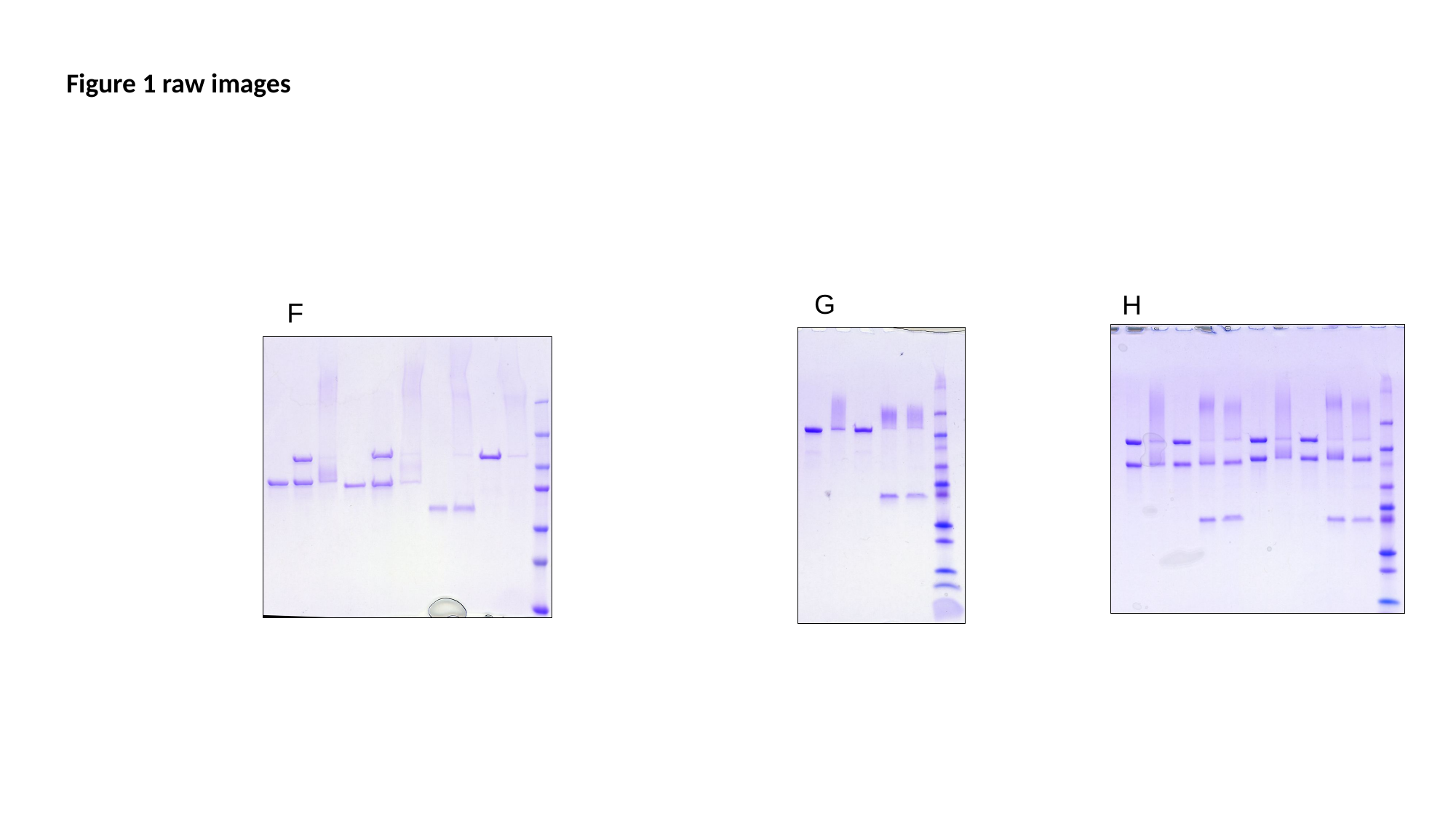

Figure 1 raw images
G
H
F

## Slide 3
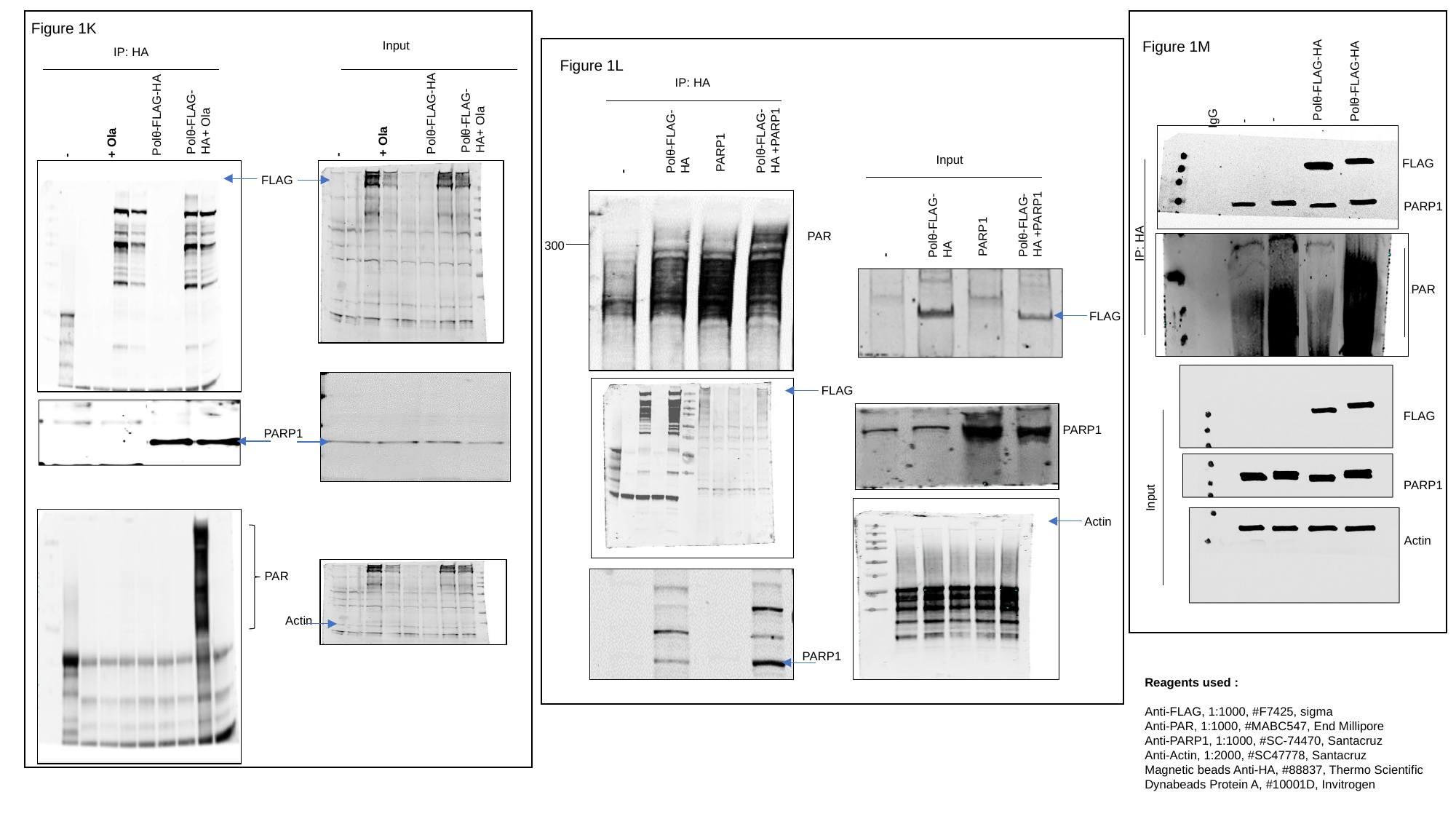

Figure 1K
Input
IP: HA
FLAG
PARP1
PAR
Actin
+ Ola
+ Ola
Polθ-FLAG-HA+ Ola
Polθ-FLAG-HA+ Ola
Polθ-FLAG-HA
Polθ-FLAG-HA
-
-
Polθ-FLAG-HA
Polθ-FLAG-HA
IgG
-
-
FLAG
PARP1
PAR
FLAG
PARP1
Actin
Figure 1M
Polθ-FLAG-
HA +PARP1
Polθ-FLAG-
HA
PARP1
-
 PAR
300
 FLAG
 FLAG
PARP1
Actin
PARP1
Figure 1L
IP: HA
Input
Polθ-FLAG-
HA +PARP1
Polθ-FLAG-
HA
PARP1
-
IP: HA
Input
Reagents used :
Anti-FLAG, 1:1000, #F7425, sigma
Anti-PAR, 1:1000, #MABC547, End Millipore
Anti-PARP1, 1:1000, #SC-74470, Santacruz
Anti-Actin, 1:2000, #SC47778, Santacruz
Magnetic beads Anti-HA, #88837, Thermo Scientific
Dynabeads Protein A, #10001D, Invitrogen

## Slide 4
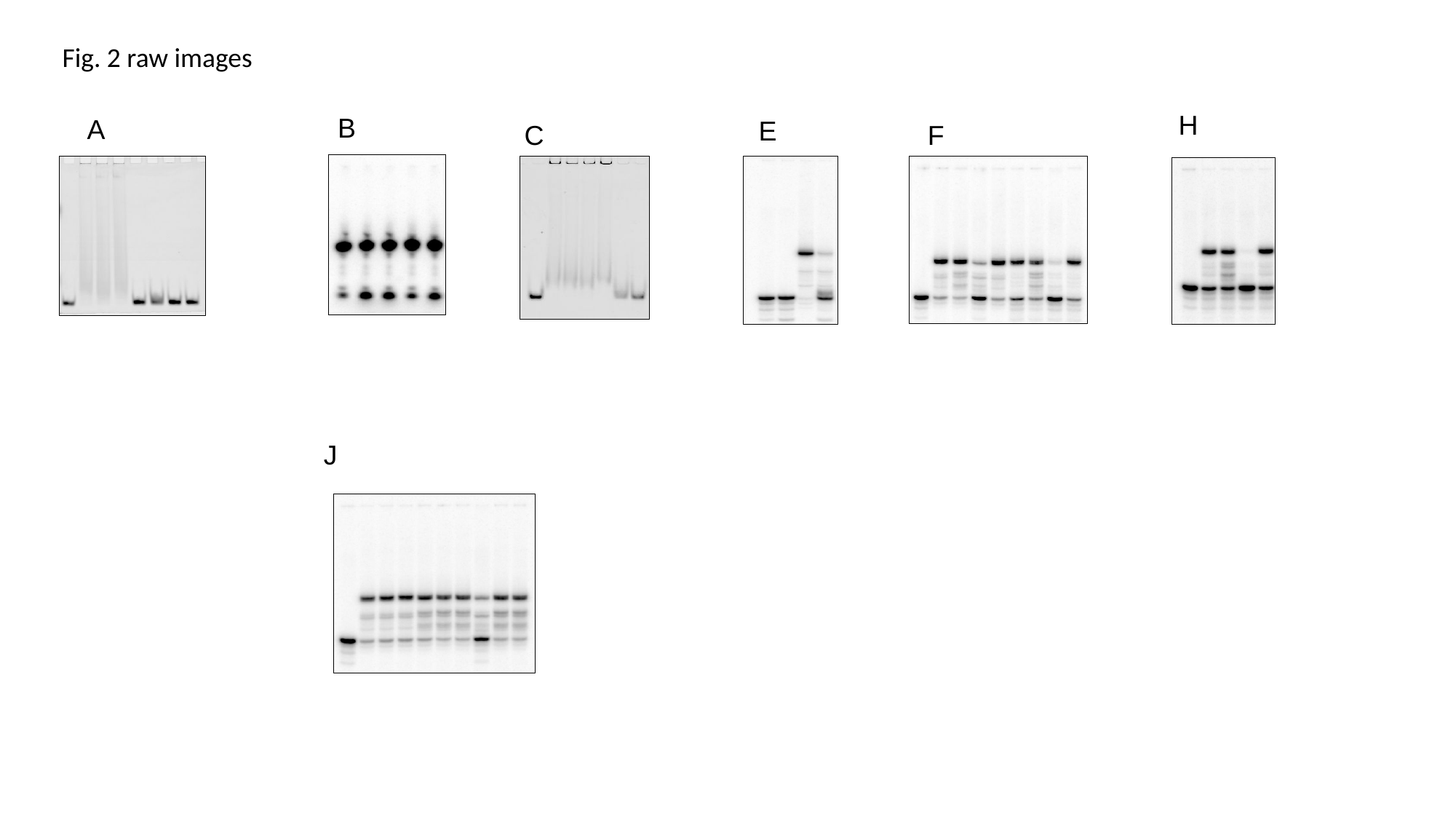

Fig. 2 raw images
H
B
A
E
F
C
J

## Slide 5
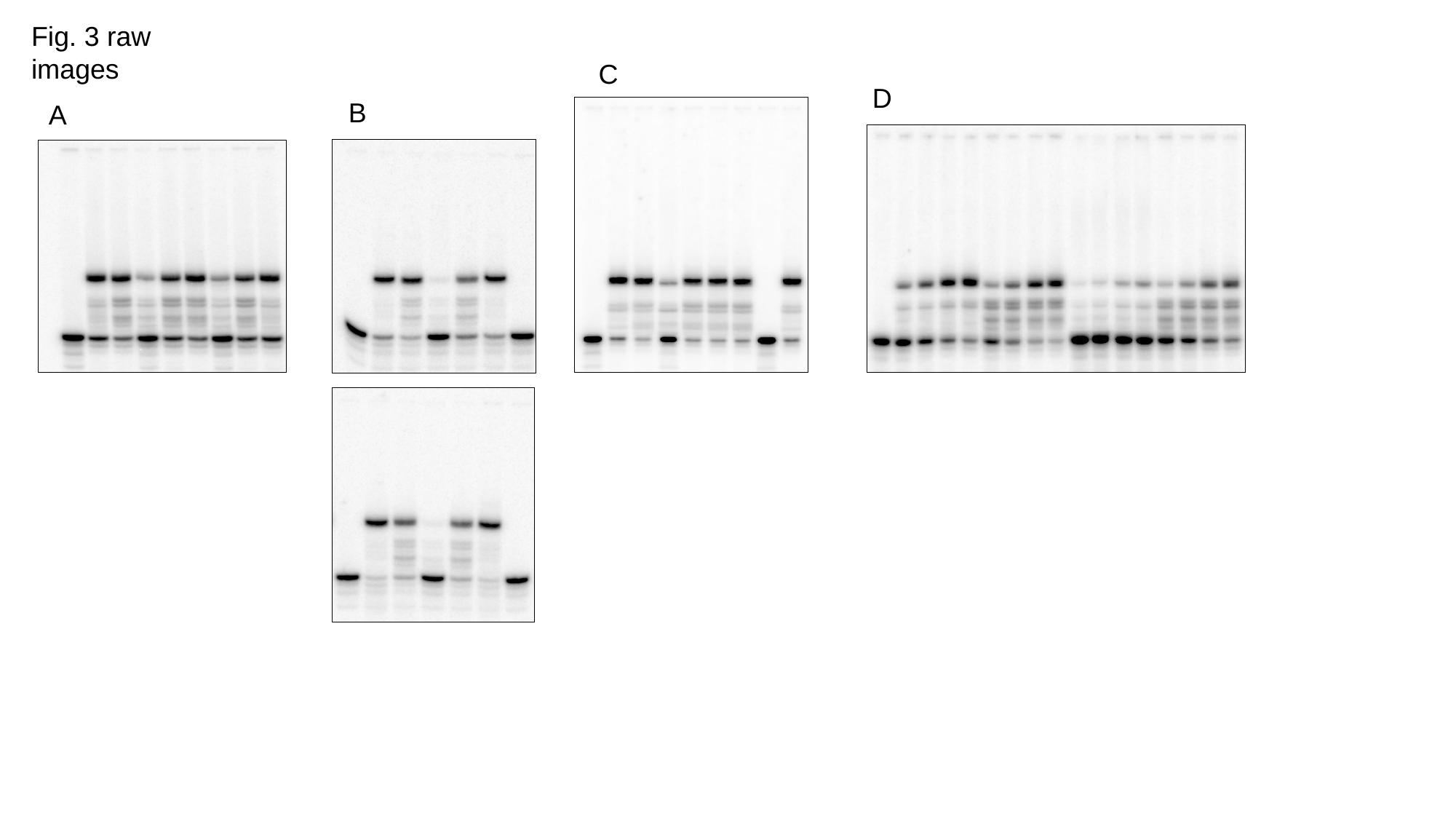

Fig. 3 raw images
C
D
B
A

## Slide 6
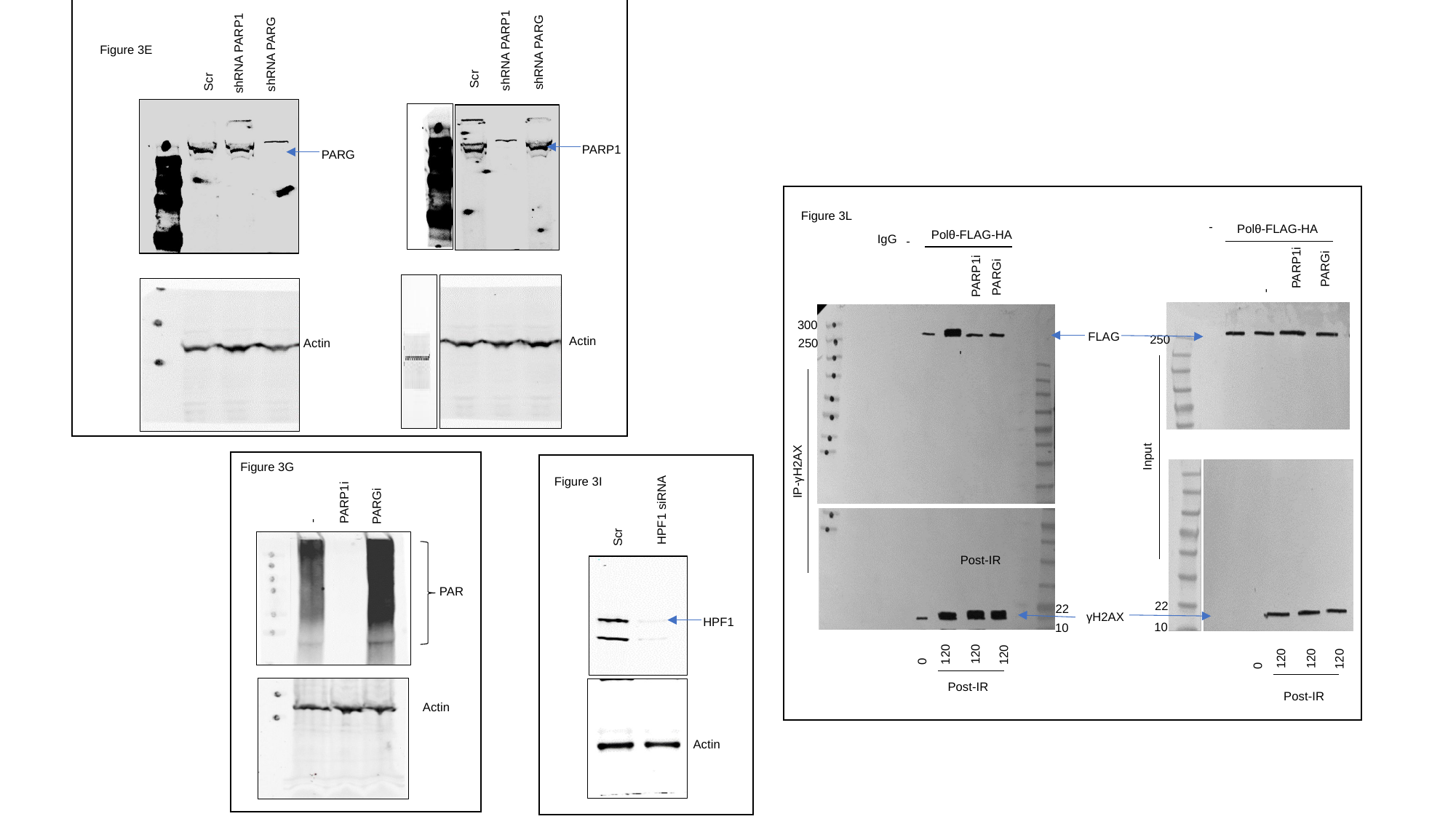

shRNA PARP1
 shRNA PARG
 shRNA PARP1
 shRNA PARG
Scr
Scr
PARP1
PARG
Actin
Actin
Figure 3E
-
Polθ-FLAG-HA
Polθ-FLAG-HA
IgG
-
PARP1i
PARGi
PARP1i
PARGi
-
300
FLAG
250
250
-
Input
IP-γH2AX
Post-IR
22
22
γH2AX
10
10
120
120
120
120
120
120
0
0
Post-IR
Post-IR
Figure 3L
PARGi
-
PARP1i
Actin
PAR
Figure 3G
HPF1 siRNA
Scr
HPF1
Actin
Figure 3I

## Slide 7
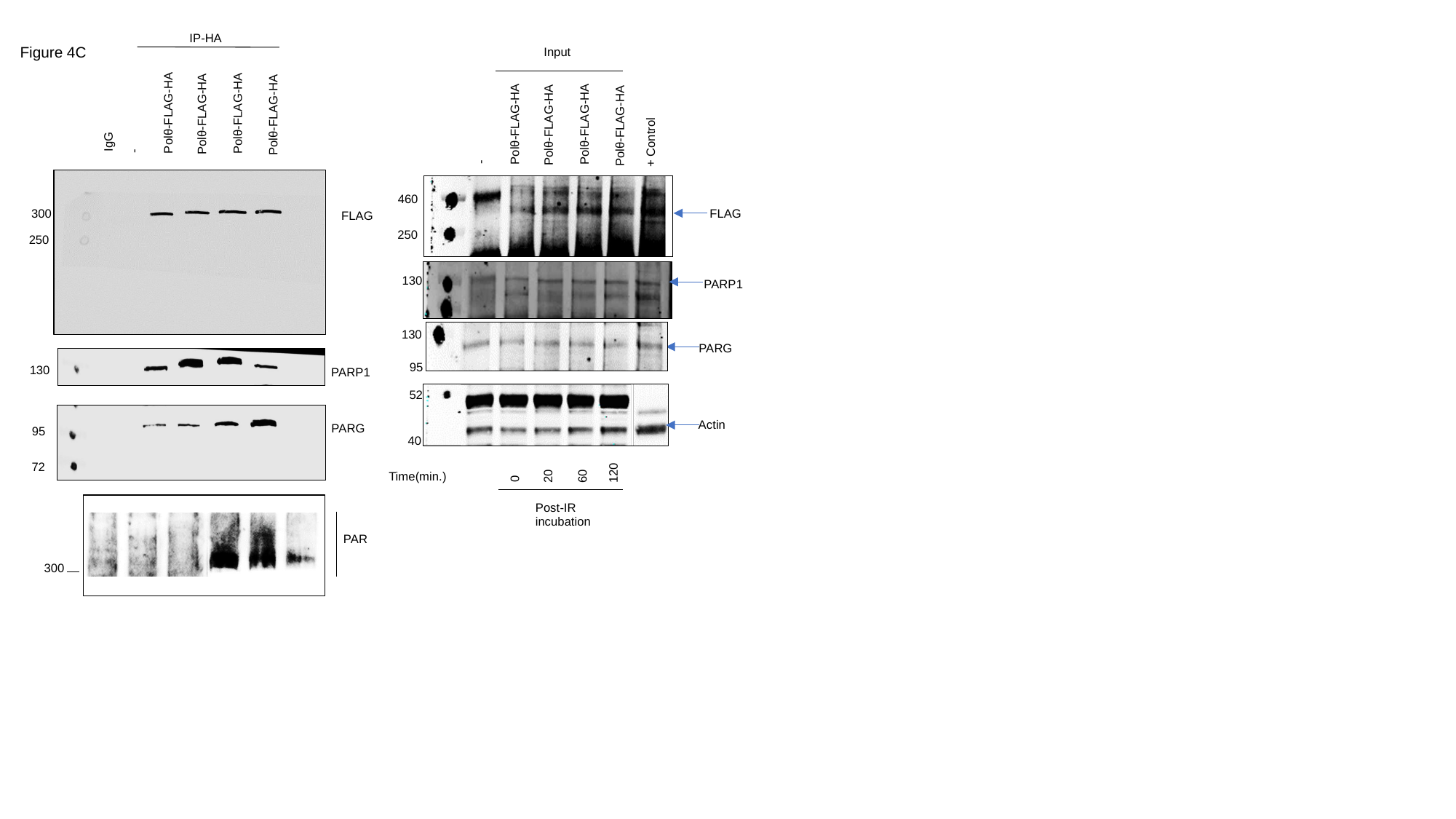

IP-HA
Input
Polθ-FLAG-HA
Polθ-FLAG-HA
Polθ-FLAG-HA
Polθ-FLAG-HA
Polθ-FLAG-HA
Polθ-FLAG-HA
Polθ-FLAG-HA
Polθ-FLAG-HA
+ Control
IgG
-
-
460
FLAG
FLAG
250
PARP1
PARG
130
PARP1
Actin
PARG
95
72
300
PAR
300
250
120
0
20
60
Time(min.)
Post-IR incubation
Figure 4C
130
130
95
52
40

## Slide 8
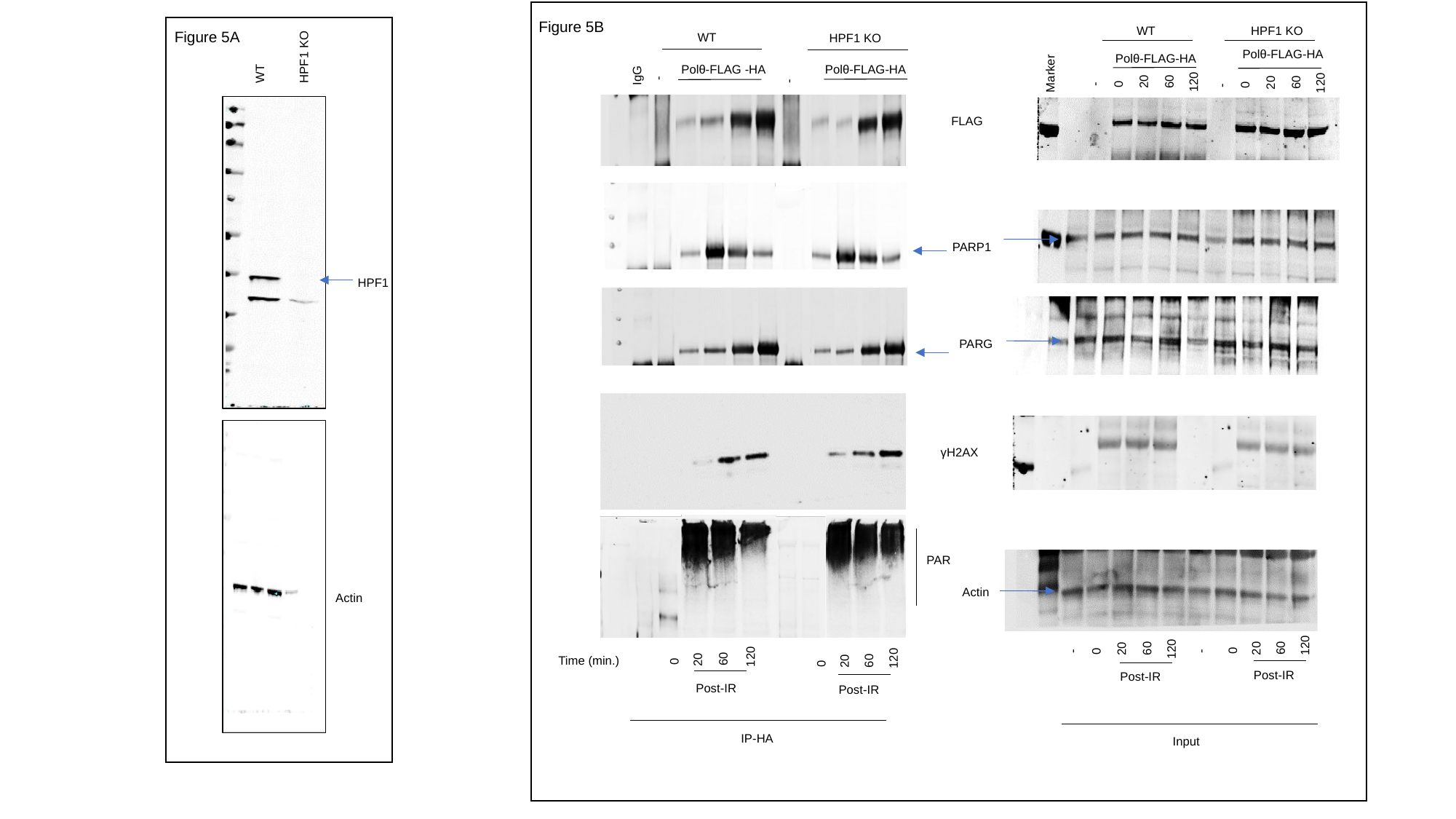

Figure 5B
Figure 5A
HPF1 KO
WT
HPF1
Actin
HPF1 KO
WT
WT
HPF1 KO
Polθ-FLAG-HA
Polθ-FLAG-HA
Polθ-FLAG-HA
Polθ-FLAG -HA
IgG
-
-
60
20
120
60
20
120
0
-
0
-
FLAG
PARP1
PARG
γH2AX
PAR
Actin
120
60
60
20
20
120
0
-
0
-
120
120
60
20
Time (min.)
60
20
0
0
Post-IR
Post-IR
Post-IR
Post-IR
IP-HA
Input
Marker

## Slide 9
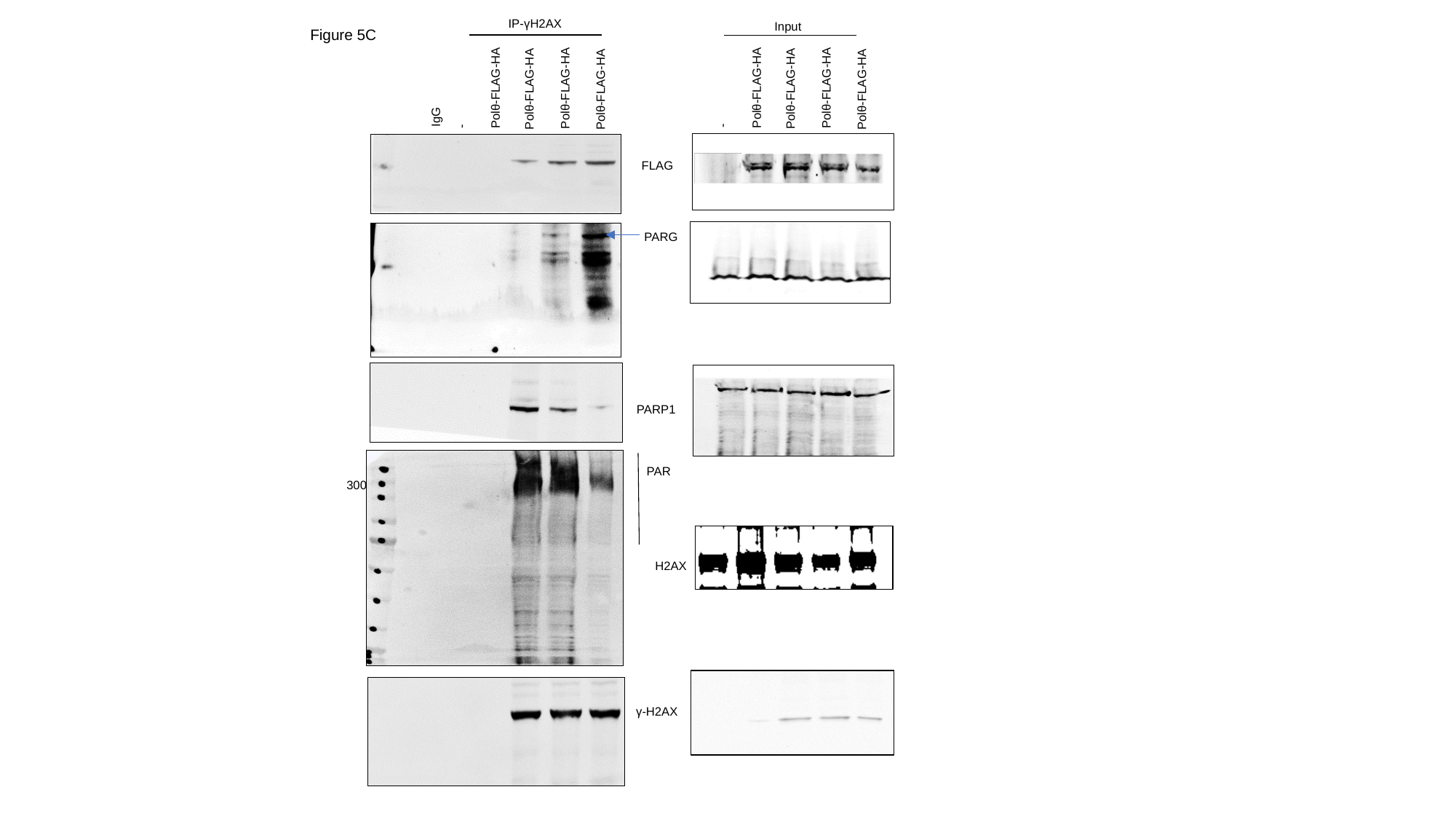

IP-үH2AX
Input
Polθ-FLAG-HA
Polθ-FLAG-HA
Polθ-FLAG-HA
Polθ-FLAG-HA
Polθ-FLAG-HA
Polθ-FLAG-HA
Polθ-FLAG-HA
Polθ-FLAG-HA
IgG
-
-
FLAG
PARG
PARP1
PAR
300
H2AX
γ-H2AX
Figure 5C

## Slide 10
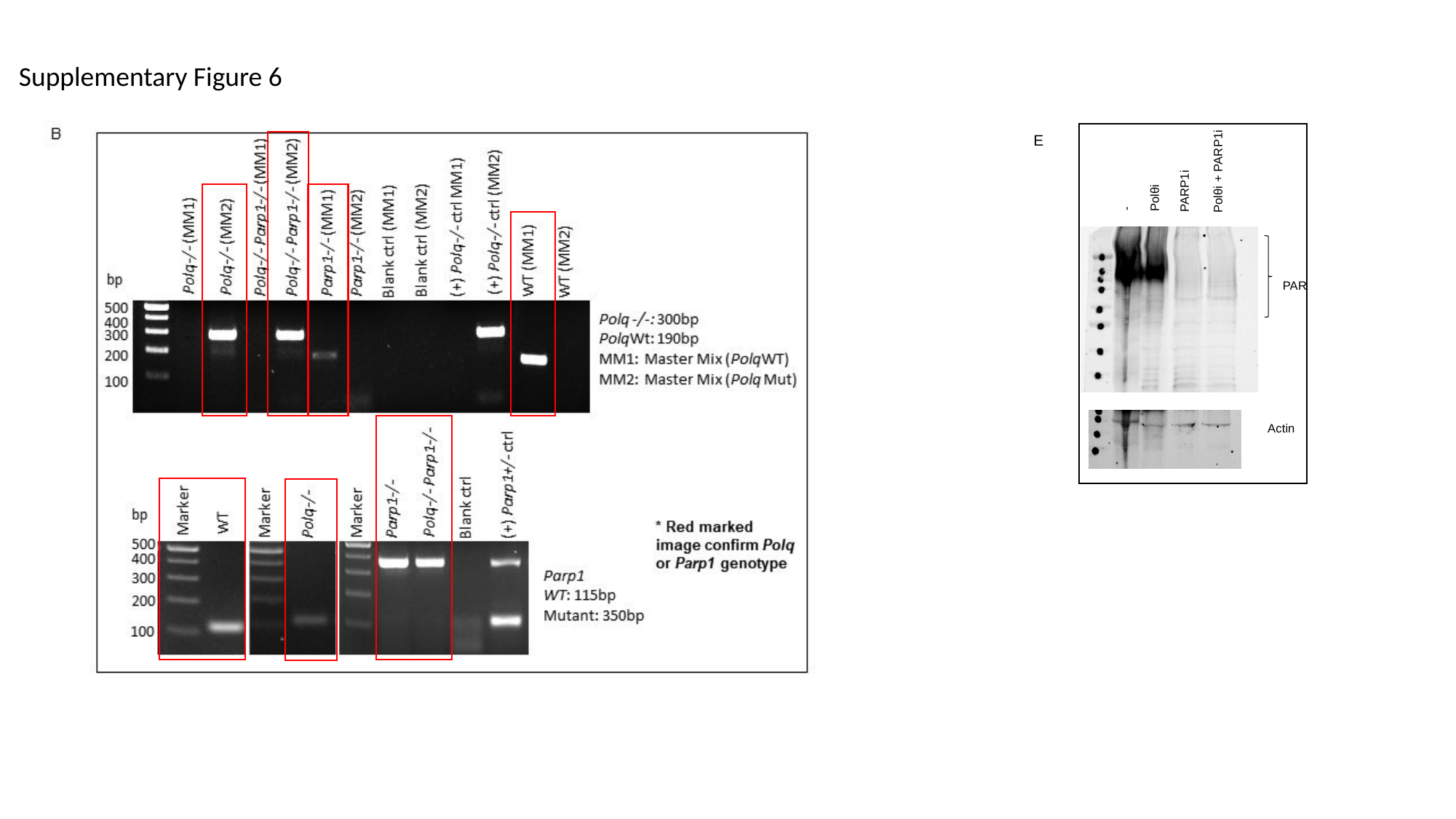

Supplementary Figure 6
Polθi + PARP1i
PARP1i
-
Polθi
PAR
Actin
E

## Slide 11
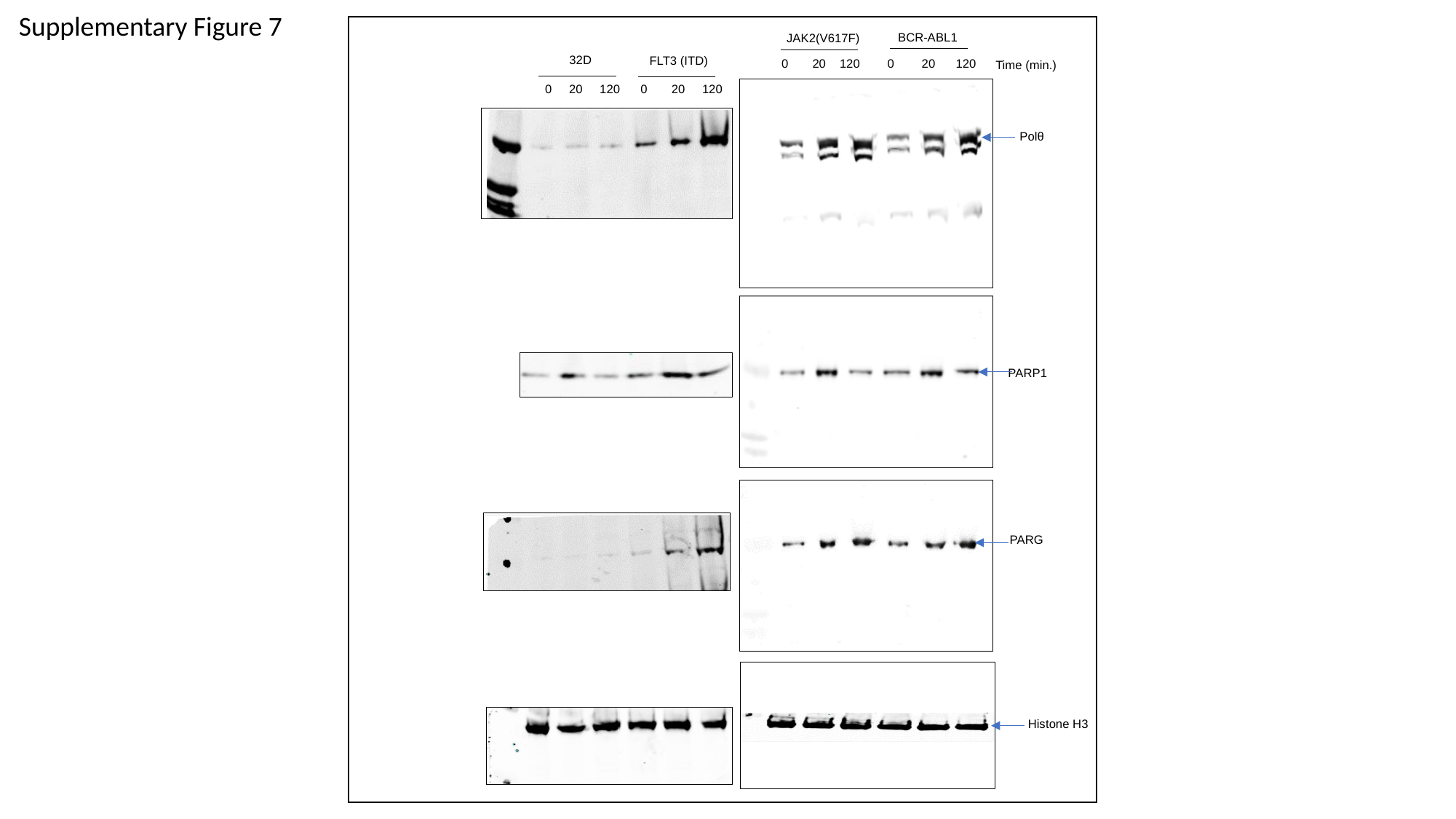

Supplementary Figure 7
BCR-ABL1
JAK2(V617F)
32D
FLT3 (ITD)
 0 20 120 0 20 120
Time (min.)
 0 20 120 0 20 120
Polθ
PARP1
PARG
Histone H3
